# Primary care and pulmonary physicians’ knowledge and practice concerning screening for lung cancer in Lebanon, a middle‐income country

**DOI:** 10.1002/cam4.3816

**Published:** 2021-03-20

**Authors:** Imad Bou Akl, Nathalie K. Zgheib, Maroun Matar, Deborah Mukherji, Marco Bardus, Rihab Nasr

**Affiliations:** ^1^ Division of Pulmonary Department of Internal Medicine American University of Beirut Faculty of Medicine Beirut Lebanon; ^2^ Department of Pharmacology and Toxicology American University of Beirut Faculty of Medicine Beirut Lebanon; ^3^ Cancer Prevention and Control Program Naef K. Basile Cancer Institute American University of Beirut Faculty of Medicine Beirut Lebanon; ^4^ Division of Hematology Oncology Department of Internal Medicine American University of Beirut Faculty of Medicine Beirut Lebanon; ^5^ Department of Health Promotion and Community Health American University of Beirut Faculty of Health Sciences Beirut Lebanon; ^6^ Department of Anatomy, Cell Biology and Physiological Sciences American University of Beirut Faculty of Medicine Beirut Lebanon

**Keywords:** cancer awareness, Lebanon, low‐dose computed tomography, lung cancer, screening

## Abstract

**Background:**

Screening for lung cancer with low‐dose computed tomography (LDCT) was shown to reduce lung cancer incidence and overall mortality, and it has been recently included in international guidelines. Despite the rising burden of lung cancer in low and middle‐income countries (LMICs) such as Lebanon, little is known about what primary care physicians or pulmonologists know and think about LDCT as a screening procedure for lung cancer, and if they recommend it.

**Objectives:**

Evaluate the knowledge about LDCT and implementation of international guidelines for lung cancer screening among Lebanese primary care physicians (PCPs) and pulmonary specialists.

**Methodology:**

PCPs and PUs based in Lebanon were surveyed concerning knowledge and practices related to lung cancer screening by self‐administered paper questionnaires.

**Results:**

73.8% of PCPs and 60.7% of pulmonary specialists recognized LDCT as an effective tool for lung cancer screening, with 63.6% of PCPs and 71% of pulmonary specialists having used it for screening. However, only 23.4% of PCPs and 14.5% of pulmonary specialists recognized the eligibility criteria for screening. Chest X‐ray was recognized as ineffective by only 55.8% of PCPs and 40.7% of pulmonary specialists; indeed, 30.2% of PCPs and 46% of pulmonary specialists continue using it for screening. The majority have initiated a discussion about the risks and benefits of lung cancer screening.

**Conclusion:**

PCPs and pulmonary specialists are initiating discussions and ordering LDCT for lung cancer screening. However, a significant proportion of both specialties are still using a non‐recommended screening tool (chest x‐ray); only few PCPs and pulmonary specialists recognized the population at risk for which screening is recommended. Targeted provider education is needed to close the knowledge gap and promote proper implementation of guidelines for lung cancer screening.

## Graphical abstract

This study highlights the gap in knowledge and practices regarding lung cancer screening among both PCPs and pulmonologists in a middle‐income country and stresses the need for better provider education about guidelines for lung cancer screening before implementing any lung cancer screening program.

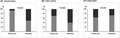

## 1 INTRODUCTION

Low and middle‐income countries (LMICs) have a rising burden of cancer death due to population aging, growth, and a decrease in deaths related to communicable diseases.^1^ Among different types of cancers, lung cancer is currently the most common worldwide and the leading cause of cancer‐related mortality (1;2). According to the World Health Organization (WHO)’s Globocan 2018 survey, Lebanon, a small middle‐income country in the East Mediterranean Region, has the highest lung cancer incidence in females and the third highest incidence in males, among countries of the Arab World.^2^ In Lebanon, lung cancer is also the second most common cancer in males, after prostate cancer, and third most common cancer in females, after breast and colorectal cancer. Lebanon has also one of the highest prevalence of smoking in the Arab world.^3^ Smoking is the leading risk factor for lung cancer,^2^ whose incidence has increased from 2005 to 2015 from 25.3 to 35.6 per 100,000 people, peaking at 37.1 per 100,000 in 2014.^4^ This increase in lung cancer rate and burden highlights the importance of prevention, through tobacco control, and early detection through the implementation of screening programs.

Early attempts to promote lung cancer screening among high risk populations started in the United States, in the early 1980 s, at the Mayo clinic, where scientists studied the use of Chest X Ray (CXR) every four months.^5^ The trial was followed by three large‐scale studies in the mid‐1980 s using CXR in combination with sputum cytology for screening high‐risk‐populations for lung cancer.^6^, ^7^, ^8^ These interventions/techniques however failed to show improvement in overall mortality in the screened group. More recently, in 2009, a prostate, lung, colorectal, and ovarian cancer screening trial with 154,901 patients used CXR as the screening tool for lung cancer. The screened and non‐screened groups had equal lung cancer mortality, as well as similar lung cancer stages, and histology results.^9^ Therefore, it was concluded that annual CXR for screening high risk patients for lung cancer is not beneficial in terms of improvement of mortality, hence the need for better screening tools.^10^

The US National Lung Screening Trial (NLST), conducted from 2002 till 2009, showed that screening with low‐dose CT (LDCT) scan of the chest reduced lung cancer mortality by 20%.^11^ This landmark study led to the implementation of a voluntary lung cancer screening program for the high risk population following the recommendations by the US Preventive Service Task Force (USPSTF),^12^ the International Association for the Study of Lung Cancer (IASLC),^13^ and the National Comprehensive Cancer Network (NCCN).^14^ As such, it is now recommended worldwide to do “annual screening for lung cancer with LDCT in adults aged 55 to 80 years who have a 30 pack‐year smoking history and currently smoke or have quit within the past 15 years. Screening should be discontinued once a person has not smoked for 15 years or develops a health problem that substantially limits life expectancy or the ability or willingness to have curative lung surgery”.^12^

The findings of the NLST have led most high‐income countries to initiate research on LDCT lung cancer screening in an effort to establish the feasibility of performing LDCT and to add to the overall knowledge base of lung cancer screening.^15^ However, LMICs have been lagging behind. In the Middle East, only Saudi Arabia has published guidelines for lung cancer screening.^16^ In addition, evaluations of the current screening practices in the Middle East are very rare with only one study in Saudi Arabia addressing primary care physician beliefs and recommendations for lung cancer screening in a single hospital,^17^ a gap that needs to be addressed if the rising lung cancer burden is to be tackled.

With the lack of dedicated screening programs in Lebanon, lung cancer screening is mostly based on the initiative of either well‐informed patients or the recommendations of well‐informed physicians, with the information being diffused through national specialty specific meetings or conferences for those who attend. Notably, in Lebanon there is no universal health care coverage for neither inpatient nor outpatient care. Only employees have social insurance coverage (in and out) with subscriptions being shared by employers and employees, while independent self‐employed individuals have the option to purchase one of the many private insurance plans with optional and usually quite costly outpatient care or remain without coverage. For the insured population with outpatient coverage, the LDCT cost is typically reimbursed, while those who have no outpatient coverage at all would be subsidized by the government only if hospitalized. Private and governmental insurance companies do not dictate a visit to a primary care physician (PCP), and a patient may hence see a pulmonary specialist directly. Therefore, the lack of structure for screening within the private and public health care systems makes the decision to screen (or not) a physician‐driven process.

To understand the current situation related to lung cancer screening guidelines in Lebanon, it is important to assess the knowledge and behaviors of physicians who are currently involved in lung cancer screening, such as primary care physicians and pulmonary specialists. This information is fundamental to plan interventions aimed at encouraging screening targeting primarily health professionals and secondarily clients (i.e., potential patients). Therefore, the aim of this study was to evaluate the knowledge and practice of meeting the international guidelines for lung cancer screening among Lebanese PCPs and pulmonary specialists.

## 2 METHODS

A cross‐sectional study was conducted using an anonymous, self‐administered one‐page paper questionnaire. The American University of Beirut Institutional Review Board (IRB) judged this study exempt from review (September 2018).

### 2.1 Participants and procedures

The questionnaire was distributed to all attendees of the meetings of the Lebanese Family Medicine Society (December 7, 2018), and Pulmonary Society (May 2, 2019). The paper questionnaire was handed at the beginning of the meeting upon registration. Participants were asked to place their answers in a sealed opaque box placed at the registration desk. There was no coercion.

### 2.2 Measurement tool

The questionnaire included items based on the USPT guidelines ^12^ and adapted from part D (Lung cancer screening) of the 2009 National Survey of Primary Care Physicians’ Cancer Screening Recommendations and Practices Colorectal and Lung Cancer Screening Questionnaire, conducted by the National Cancer Institute (NCI) in collaboration with the Center for Disease Control and Prevention (CDC) and Agency for Healthcare Research and Quality (AHRQ).^18^

A section assessed respondents’ knowledge of the proper screening tools for lung cancer, and the population at risk for which screening is indicated. Five questions assessed the physicians’ current behavior (i.e., recommending lung cancer screening), and three inquired on their experience with patients asking for screening of three common cancer types: breast, colon, and lung. The questions were validated by a group of physician colleagues for content and clarity.

The questionnaire was in English, as most physicians in Lebanon typically learn and speak English, and medical conferences in Lebanon are typically held in English. There were no personal nor institutional identifiers throughout the whole survey. See Appendix S1 for the full questionnaire.

### 2.3 Statistical analysis

Responses were manually entered into SPSS (v. 24, IBM, Armonk, NY, USA). Data are presented as numbers with frequencies, and proportions between the two physician specialties were compared using chi‐square tests. A Z‐ test was also used for comparison of proportions of some of the respondents’ characteristics. A *P*‐value of less than 0.05 was considered statistically significant.

## 3 RESULTS

### 3.1 Participants

The meetings of the Lebanese Family Medicine Society and of the Pulmonary Society were respectively attended by 150 PCPs and 100 pulmonary specialists. In the first meeting we recruited 47 PCPs (30% response rate). Of these, 29 (70.7%) were family medicine specialists. Of 100 estimated attendees of the pulmonary medicine meeting, 62 participated in the study (60% response rate). In Lebanon there is approximately 170 registered pulmonary specialists, and 178 PCPs.

As seen in Table 1, the majority of participants were practicing physicians working in Lebanon. Both trainees and practicing physicians were included in the data analysis. There were no significant differences in the years of experience, as about half of all participants (53.7% of the primary care and 51.7% of the pulmonary physicians) had been practicing for more than 10 years (*p* = 0.111). Both groups included a good mix of physicians with and without academic affiliations with a significantly larger share of the PCPs having academic affiliations when compared to pulmonary specialists [58.1% vs. 28.6%; *p* = 0.003].

**TABLE 1:** Comparison between characteristics^a^ of primary care versus pulmonary physicians

Specialty	Primary care	Pulmonary	*P‐value*
Number	47	62	
Characteristic	*N* (%)	*N* (%)	
Career stage			
Practicing physician	34 (82.9)	52 (83.9)	0.100
Trainee	7 (17.1)	10 (16.1)	
Years of practice			
<5 years	15 (36.6)	14 (23.3)	0.111
5–10 years	4 (9.8)	15 (25.0)	
>10 years	22 (53.7)	31 (51.7)	
Country of practice			
Lebanon	22 (84.6)	14 (88.9)	0.584
Outside Lebanon	2 (7.7)	0 (0)	
Both	2 (7.7)	3 (11.1)	
Affiliated with an academic institution			
YES	25 (58.1)	17 (28.3)	**0.003**
NO	18 (41.8)	43 (71.7)	

^a^ Answers may not add up to the total number due to some missing data.

### 3.2 Physicians’ knowledge about lung cancer screening

As shown in Table 2, 55.8% of PCPs recognized that a CXR is an ineffective screening tool for lung cancer in asymptomatic patients compared to only 40.7% of pulmonary specialists, a difference between the two groups that was not significant. Similarly, 73.8% of PCPs recognized LDCT as a very effective tool for lung cancer screening compared to only 60.7% of the pulmonary specialists. Significantly more PCPs recognized that CXR is not indicated for any risk population while more than half of the pulmonary specialists would use CXR to screen for both proposed risk population scenarios in the survey. Regarding the eligibility criteria for LDCT, 23.4% of PCPs and significantly less pulmonologists (14.5%) correctly recognized the population at risk for which lung cancer screening is indicated (i.e. the 55 y.o. subject with history of 30 pack year and who has quit smoking only 2 years before).

**TABLE 2:** Comparison between primary care and pulmonary physicians of their knowledge^a^ concerning screening for lung cancer

Specialty	Primary care	Pulmonary	*P‐value*
Number^b^	47	62	
Question	*N* (%)	*N* (%)	
How effective is the below screening procedure in reducing lung cancer mortality in asymptomatic patients that are current heavy smokers and aged 60 years and older?			
Chest X‐ray			
Very effective	4 (9.3)	11 (18.6)	0.212
Somewhat effective	14 (32.6)	24 (40.7)	
Not effective	24 (55.8)	24 (40.7)	
Don't know	1 (2.3)	0 (0)	
Low dose ration CT			
Very effective	31 (73.8)	37 (60.7)	0.090
Somewhat effective	8 (19.0)	23 (37.7)	
Not effective	1 (2.4)	1 (1.6)	
Don't know	2 (4.8)	0 (0)	
For which of the below scenarios would you screen for lung cancer on a healthy asymptomatic patient with no history of lung disease nor family history of lung cancer using…?			
Chest X‐ray			
58 y.o, history of 20 pack years, currently smoker	2 (4.3)	3 (4.9)	**0.029**
55 y.o, history of 30 pack years, has quit smoking 2 years	0 (0)	1 (1.6)	
Both of the above	14 (29.8)	34 (55.7)	
None of the above	31 (66.0)	23 (37.7)	
Low radiation dose spiral CT			
58 y.o, history of 20 pack years, currently smoker	6 (12.8)	2 (3.2)	**0.049**
55 y.o, history of 30 pack years, has quit smoking 2 years	11 (23.4)	9 (14.5)	
Both of the above	27 (57.4)	39 (62.9)	
None of the above	3 (6.4)	12 (19.4)	

^a^ Highlighted cells indicate the correct answers.

^b^ Answers may not add up to the total number due to some missing data.

Looking at differences in physician's knowledge based on the type of practice they have, 64% of physicians in academic institutions recognized that a CXR is ineffective, and 76.7% recognized that CXR is not indicated for any risk population compared to 32.7% and 25%, respectively, in physicians in non‐academic settings (Table S1 **)**. A difference that is statistically significant and may explain why more PCPs recognized the limitation of CXR as a screening tool when compared to pulmonary specialists as significantly more of them were in an academic type of practice. There were no other statistically significant differences between physicians practicing in academic and non‐academic setting in any of the other questions.

Notably, the majority of physicians from both specialties answered that they knew the approximate cost of both CXR [95.7% for primary care and 93.4% for pulmonary physicians; *p* = 0.698] and LDCT, though with a lesser proportion when compared to the cost of CXR [73.9% for primary care and 75.5% for pulmonary physicians; *p* = 0.821 knew the cost of LDCT]. In addition, a LDCT is available in the area of practice of most respondents [77.8% for primary care and 85.0% for pulmonary physicians; *p* = 0.447] (Data not shown).

### 3.3 Physicians’ practice concerning screening for lung cancer

When asked about the screening practices over the preceding 12 months (Table 3), 30.2% of primary care physicians and 46% of pulmonologists had ordered a CXR. Respectively 63.6% and 71% have ordered LDCT, but the differences were not significant. Significantly more pulmonary physicians (56.7%) discussed the results of a CXR with a patient who self‐referred for the procedure than primary physicians (44.4%). A similar percentage of pulmonary physicians (69.4%) and primary care physicians (57.8%) discussed the results of LDCT with a patient who self‐referred for the procedure. Both physician groups had initiated a discussion about the risks and benefits of lung cancer screening with similar frequency (81.8% for primary care vs. 77.4% for pulmonary specialists).

**TABLE 3:** Comparison between primary care and pulmonary physicians of their practice^a^ concerning screening for lung cancer

Specialty	Primary care	Pulmonary	*P‐value*
Number^b^	47	62	
QuestioN	*N* (%)	*N* (%)	
For the past 12 months, for an asymptomatic patient, did you ever:			
Order a chest X ray for lung cancer screening?			
YES	13 (30.2)	28 (46.7)	0.106
NO	30 (69.8)	32 (53.3)	
Don't know	‐	‐	
Order a low radiation dose spiral CT for lung cancer screening?			
YES	28 (63.6)	44 (71.0)	0.527
NO	16 (36.4)	18 (29.0)	
Don't know	‐	‐	
Discuss with a patient who had self‐referred for the procedure, the results of a chest X ray?			
YES	20 (44.4)	40 (56.7)	**0.035**
NO	19 (42.2)	18 (80.0)	
Don't know	6 (13.3)	2 (3.3)	
Discuss with a patient who had self‐referred for the procedure, the results of a low radiation dose spiral CT?			
YES	26 (57.8	43 (69.4)	0.158
NO	19 (42.2)	17 (27.4)	
Don't know	0 (0)	2 (3.2)	
Initiate a discussion about the risks and benefits of lung cancer screening?			
YES	36 (81.8)	48 (77.4)	0.472
NO	8 (18.2)	12 (19.4)	
Don't know	0(0)	2 (3.2)	

^a^ Highlighted cells indicate potentially good practice.

^b^ Answers may not add up to the total number due to some missing data.

### 3.4 Patient‐triggered screening for breast, colon, and lung cancer

**FIGURE 1:**
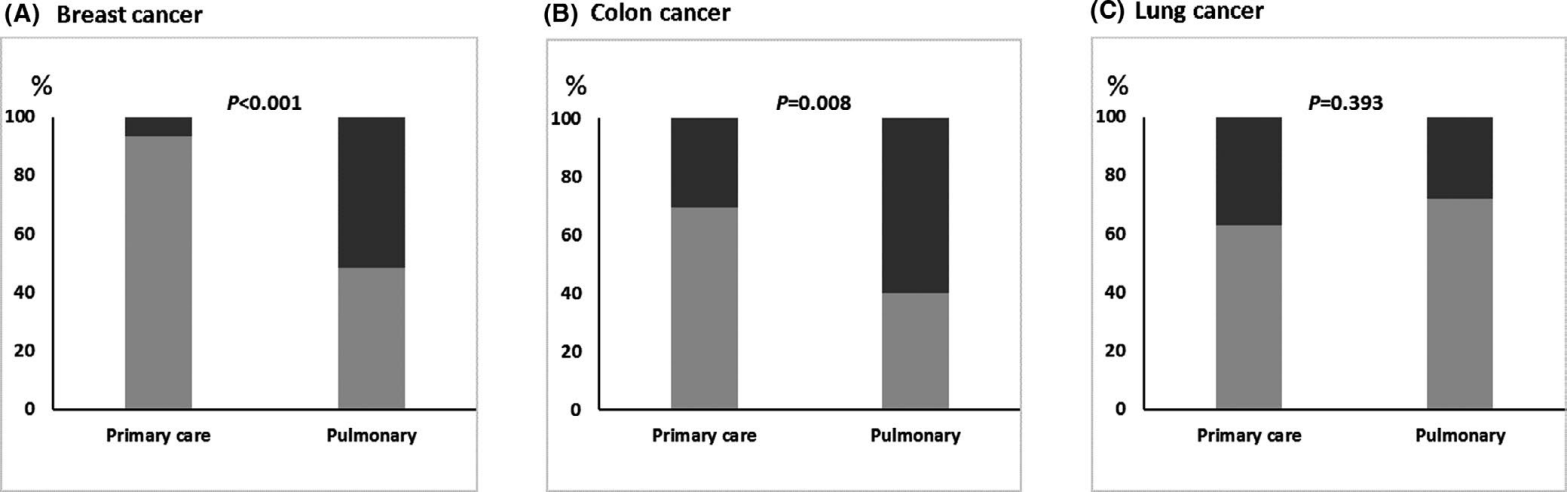
Comparison between primary care and pulmonary physicians concerning the frequency of whether their patients have asked in the past 12 months if they can or should be screened for Breast cancer (A), colon cancer (B) or lung cancer (C)

As shown in Figure 1, physicians operating in primary care settings reported that patients asked referrals mostly for breast cancer screening (93.3%), followed by colon cancer (69.6%), and lastly by lung cancer screening (62.8%). The proportions were significantly different for breast cancer (*p* < 0.001) and colon cancer (*p* = 0.008), but not for lung cancer (*p* = 0.393).

## 4 DISCUSSION

In this study, we sought to understand and compare the current knowledge and implementation of lung cancer screening guidelines among PCPs and pulmonary specialists, who are most likely to initiate the lung cancer screening process. This study is the first of its kind in Lebanon and the second in the Middle East.^17^ Four important findings regarding knowledge and practice may be gleaned from the results of this survey. First, there are gaps in knowledge regarding the proper screening method for lung cancer, as a significant proportion of providers (44.2% of PCPs and 59.3% of pulmonologists) did not recognize that CXR is an ineffective tool for lung cancer screening. In addition, a significant percentage of physicians would still prescribe CXR to screen for lung cancer (34% of PCP, and 62.3% of pulmonologists) instead of LDCT. This gap is especially pronounced in the non‐academic practices of both groups, with as many as 75% of them would use CXR as a screening tool, and only 32% of them found it as an ineffective tool for screening. In practice, CXR is still being used for screening by as much as 30.2% of PCPs and 46.7% of pulmonologists. Second, although a good number of providers considered LDCT to be an effective tool for lung cancer screening (73.8% of PCPs and 60.7% of pulmonologists), a very low number recognized the eligibility criteria for LDCT (23.4% of PCP and 14.5% of pulmonologists). Third, a smaller share of pulmonary specialists recognized the high‐risk population for which LDCT is indicated and significantly more of them would use CXR to screen and have discussed the CXR findings of a self‐referred patient for lung cancer screening. Last, patient‐triggered screening in the primary care setting was highest for breast at 93.3%, followed by colorectal (69.6%), and lung (62.8%) cancers, which is aligned with trends in cancer screening in Lebanon.^19^

Despite multiple studies showing the limited efficacy of CXR as a screening tool for lung cancer,^6^, ^7^, ^8^ and the recent NLST trial ^11^ showing the clear efficacy and superiority of LDCT in reducing mortality in lung cancer screening, CXR is still used for cancer screening. This finding is consistent with other US studies reporting that PCPs are still using CXR for lung cancer screening, albeit at a much lower frequency of around 20%.^20^, ^21^ In addition, Couraud et al. ^22^ found that both PCPs and pulmonary specialists in France are also still using CXR, but unlike our findings, the pulmonary specialists ordered it less than the PCPs. The large number of studies using CXR as a screening tool for lung cancer extending from 1983 till 2009 may have left the impression that it is a useful but yet to be proven screening method (5–9;11). This may explain why it is still in use for screening among physicians and especially among physicians in non‐academic type of practice which may have a more limited access to the latest international recommendations and guidelines. In this case, the national order of physicians and specialized societies should play an important role in promoting these guidelines among their members.

Unlike other cancers for which screening encompasses an entire population after a certain age, the guidelines for lung cancer screening indicate that the high‐risk population is people above 55 years of age, who have a 30 pack year smoking history, and who are currently smokers, or quit smoking 15 years ago or less. It might be that these recommendations are not clearly understood, as a significant number of PCPs and pulmonologists failed to correctly identify the population at risk. The slightly higher number of PCPs who recognized the population at risk in Lebanon may reflect better knowledge and adherence to guidelines among PCPs than among pulmonary specialists. The limited knowledge of the eligibility criteria for lung cancer screening detected in this study is a common finding in many other US‐based studies addressing this issue.^21^, ^22^, ^23^ Notably, the percentage of physicians recognizing the eligibility criteria was much higher in the US. For example with Triplette et al.,^23^ 69% of providers correctly assessed eligibility in at least three of the four scenarios presented, and with Ersek et al.,^21^ 48–78% correctly recommended screening depending on the proposed vignette.

Results from this study also show that most providers initiated a discussion about the risks and benefits of lung cancer screening (81.8% of PCPs and 77.4% of pulmonologists) and most of them ordered LDCT for lung cancer screening in the last 12 months (63.6% of PCPs and 71.0% of pulmonologists). Also these results are aligned with those reported in other studies, conducted in the United States, showing that around 80% of providers initiate screening and discussion about lung cancer (21;24). For example, Rajupet et al. ^24^ found that although PCPs were less comfortable with screening than specialists, PCPs and specialists were equally likely to recommend LDCT scans for lung cancer screening. Others, such as Henderson et al.,^25^ found that significantly more pulmonologists reported ordering LDCT.

Patient‐triggered screening reflects differentials in patient awareness regarding cancer screening among the three most prevalent types of cancer. Results from this survey show that in the PCP setting, physicians reported that patients requested referrals mostly for breast cancer screening (93.3%), while colon and lung cancer screening referrals were less frequently requested (69.6% and 62.8% respectively). This difference may reflect a higher level of awareness among patients of the benefits of breast cancer screening, as opposed to colon or lung cancer screening. This discrepancy may be due to the effect of national campaigns for breast cancer screening, sponsored by the Ministry of Public Health,^26^ which have been taking place in Lebanon yearly since 2002. This long‐lasting national mass media campaign has consistently highlighted the importance of early detection of breast cancer and is complemented by a policy, which allows women to undertake free mammograms for three months (October‐December); this is intended to benefit mostly uninsured, low‐income patients living in remote areas.^26^ Additionally, there is a Breast Cancer National Task Force (BCNTF) which produced national guidelines for breast cancer screening based on available local epidemiological data, which are clearly promoted among health professionals.^27^ For colon cancer awareness campaigns promoting screening for early detection are still in their infancy. Only in March 2019, the Ministry of Public Health launched the first National Colon Cancer Awareness Campaign focusing on raising awareness on colon cancer and encouraging eligible citizens to undergo Fecal Immunochemical Test (FIT) screening. On the day of the launch of the campaign, 1000 FITs were made available for free at a specific public hospital for those who benefit from the Ministry of Public Health's services.^28^, ^29^ In comparison, and as previously mentioned, there are no national or local lung cancer screening programs. A successful lung cancer screening program in Lebanon should also address patient‐related barriers to receiving LDCT such as cost, lack of awareness, stigma related to being a smoker, fatalistic beliefs, and fear of radiation exposure. ^30^, ^31^ Research studies investigating the relevance of these barriers among the Lebanese population are still needed, as our study only targeted physicians’ knowledge and practices. The difference may be partially due to the lower number of individuals eligible for screening for lung cancer.

We believe there are several lessons to be learned from this study for the implementation of lung cancer screening in Lebanon. First, a provider‐centred education for PCPs and pulmonary specialists is needed, as there is a lack of adequate screening knowledge and understanding of the adequacy of screening tools, and eligibility for screening. Knowledge and awareness of lung cancer screening guidelines are associated with increased utilization of LDCT for screening and with an increased rate of discussion about the risks and benefits of screening with at risk patients.^32^ These educational activities can be spearheaded by Lebanese pulmonary and family medicine societies through educational conferences or Continous Medical Education (CME) activities targeting their respective physician populations and more importantly targeting physicians practicing in non‐academic settings. Local guidelines developed by these two societies, would also help raise this awareness and drive lung cancer screening. With the physicians clearly instructed about and aware of current guidelines, lung cancer screening programs would become more effective and efficient.

## 5 LIMITATIONS

This study entails a number of limitations. The sample was based on convenience, as surveyed physicians were attending the main national meetings related to their specialty. Hence, the results cannot be generalizable to all registered pulmonologists or primary care physicians, or to other specialties. Nonetheless, one might expect that physicians who do not attend such conferences may have even more limited knowledge of the latest guidelines regarding lung cancer screening. The sample includes also some response bias, as only a proportion of the attendees voluntarily returned the questionnaire, even though our overall response rate (44%) but falls within the range of other physician surveys on lung cancer screening (21;24;25). Additionally, all responses are self‐reported and some answers may have been influenced by recall bias.

## 6 CONCLUSION

This study shows that although PCPs and pulmonologists commonly discuss the risks and benefits of lung cancer screening with their patients, there is still a gap in knowledge regarding the current guidelines for lung cancer screening. It also shows the similarities and differences between PCPs and pulmonologists regarding knowledge and practices towards lung cancer screening. These results stress the need for better provider education about guidelines for lung cancer screening before implementing any lung cancer screening program. Once the guidelines are clear among providers who recommend the right type of screening, lung cancer screening programs can be initiated, as referral is an important barrier to access to healthcare services. Once these programs are implemented, more studies should assess barriers for screening among physicians and patients, in order to develop strategies aimed at encouraging lung cancer screening.

## CONFLICT OF INTEREST

The authors declare no competing financial interests.

## AUTHOR CONTRIBUTIONS

Imad Bou Akl, Maroun Matar, and Nathalie K. Zgheib: collecting and analyzing data, and writing the manuscript. Nathalie K. Zgheib, Deborah Mukherji, Marco Badrus, and Rihab Nasr: designing and driving the study, analyzing data, and critical review of the manuscript.

## Supporting information

Table S1

Appendix S1

## Data Availability

The authors confirm that the data supporting the findings of this study are available within the article and its supplementary materials.
