# Supplementary material for: Primary care and pulmonary physicians’ knowledge and practice concerning screening for lung cancer in Lebanon, a middle‐income country
Source: Cancer Med. 2021 Mar 20;10(8):2877–84. doi: 10.1002/cam4.3816 (PMC8026943; doi:10.1002/cam4.3816)
Supplement: Supplementary file 1 — Table S1 [file CAM4-10-2877-s002.docx]

| **Specialty** |  | **Academic** | **non academic** | ***P-value*** |
| --- | --- | --- | --- | --- |
| **Number^2^** |  | **43** | **60** |  |
| **Question** |  | **N (%)** | **N (%)** |  |
| **How effective is the below screening procedure in reducing lung cancer mortality in asymptomatic patients that are current heavy smokers and aged 60 years and older?** | | | | |
| **Chest X-ray** | Very effective | 6 (15.4) | 9 (16.4) | **0.014** |
|  | Somewhat effective | 8 (20.5) | 27 (49.1) |  |
|  | Not effective | 25 (64.1) | 18 (32.7) |  |
|  | Don’t know | 0 (0) | 1 (0) |  |
| **Low dose ration CT** | Very effective | 29 (69.0) | 33 (62.3) | 0.599 |
|  | Somewhat effective | 12 (28.6) | 17 (32.1) |  |
|  | Not effective | 1 (2.4) | 1 (1.9) |  |
|  | Don’t know | 0 (0) | 2 (3.8) |  |
| **For which of the below scenarios would you screen for lung cancer on a healthy asymptomatic patient**  **with no history of lung disease nor family history of lung cancer using…?** | | | | |
| **Chest X-ray** | 58 y.o, history of 20 pack years, currently smoker | 1 (2.3) | 4 (7.1) | **0.000** |
|  | 55 y.o, history of 30 pack years, has quit smoking 2 years | 1 (2.3) | 0 (0) |  |
|  | Both of the above | 8 (18.6) | 38 (67.9) |  |
|  | None of the above | 33 (76.7) | 14 (25) |  |
| **Low radiation dose spiral CT** | 58 y.o, history of 20 pack years, currently smoker | 4 (9.3) | 4 (7.1) | 0.071 |
|  | 55 y.o, history of 30 pack years, has quit smoking 2 years | 11 (25.6) | 4 (7.1) |  |
|  | Both of the above | 23 (53.5) | 38 (67.9) |  |
|  | None of the above | 5 (11.6) | 10 (17.9) |  |

**Supplemental Table 1.** Comparison between physicians practicing in academic and non-academic institutions of their **knowledge^1^** concerning screening for lung cancer

**^1^**Highlighted cells indicate the correct answer

**^2^**Answers may not add up to the total number due to some missing data
