# Supplementary material for: Primary care and pulmonary physicians’ knowledge and practice concerning screening for lung cancer in Lebanon, a middle‐income country
Source: Cancer Med. 2021 Mar 20;10(8):2877–84. doi: 10.1002/cam4.3816 (PMC8026943; doi:10.1002/cam4.3816)
Supplement: Supplementary file 2 — Appendix S1 [file CAM4-10-2877-s001.doc]

**APPENDIX I.** Survey questionnaire

You are a:  Trainee  Practicing physician

If you are a **practicing physician**, please fill the below information:

**Medical specialty**:  Primary Care Physician  Pulmonary Sepcialist  Other

**Years of Practice**:  <5 years 5-10 years  >10 years **Country of practice**:  Lebanon  Outside Lebanon

**Type of Practice:**  Solo private  Group private  Affiliated with an academic institution

| **How effective do you believe the screening procedures listed below are in reducing lung cancer mortality in the following asymptomatic patients that are current heavy smokers and aged 60 years and older?** | **Very effective** | **Somewhat effective** | **Not effective** | **Don’t know** |
| --- | --- | --- | --- | --- |
| **Chest X ray** |  |  |  |  |
| **Low radiation dose spiral CT:** |  |  |  |  |

For which of the below scenarios would you screen for lung cancer on a healthy asymptomatic patient

with no history of lung disease nor family history of lung cancer using a **Chest X ray?**

1. 58 y.o, history of 20 pack years, currently smoker
2. 55 y.o, history of 30 pack years, has quit smoking 2 years ago
3. Both of the above
4. None of the above

For which of the below scenarios would you screen for lung cancer on a healthy asymptomatic patient

with no history of lung disease nor family history of lung cancer using **Low radiation dose spiral CT?**

1. 58 y.o, history of 20 pack years, currently smoker
2. 55 y.o, history of 30 pack years, has quit smoking 2 years ago
3. Both of the above
4. None of the above

| **For the past 12 months, for an asymptomatic patient, did you ever:** | **YES** | **NO** | **Don’t know** |
| --- | --- | --- | --- |
| Order a **chest X ray** for lung cancer screening? |  |  |  |
| Order a **low radiation dose spiral CT** for lung cancer screening? |  |  |  |
| Discuss with a patient who had self-referred for the procedure, the results of a **chest X ray**? |  |  |  |
| Discuss with a patient who had self-referred for the procedure, the results of a **low radiation dose spiral CT**? |  |  |  |
| Initiate a discussion about the risks and benefits of lung cancer screening? |  |  |  |

| **For the past 12 months, did any of your patients ask if they can or should be screened for:** | **YES** | **NO** | **If YES, estimate how many?** |
| --- | --- | --- | --- |
| **Breast** cancer? |  |  | ------------ |
| **Colon** cancer? |  |  | ------------ |
| **Lung** cancer? |  |  | ------------ |

Do you know the approximate cost of a chest X ray? YES  NO

Do you know the approximate cost of a low radiation dose spiral CT? YES  NO

Is there a low radiation dose spiral CT in your area of practice? YES  NO  Do not know

**Comments**: ----------------------------------------------------------------------------------------
